# Alkali metal impact on structural and phonon properties of Er^3+^ and Tm^3+^ co-doped MY(WO_4_)_2_ (M = Li, Na, K) nanocrystals†

**DOI:** 10.1039/c7ra10706d

**Published:** 2018-01-10

**Authors:** P. Ropuszyńska-Robak, P. E. Tomaszewski, L. Kępiński, L. Macalik

**Affiliations:** Wrocław University of Economics, Faculty of Engineering and Economy, Department of Bioorganic Chemistry Komandorska 118/120 53-345 Wrocław Poland; Institute of Low Temperature and Structure Research, Polish Academy of Sciences Okólna 2 50-422 Wrocław Poland L.Macalik@int.pan.wroc.pl

## Abstract

The Pechini and microwave-assisted hydrothermal syntheses of nanocrystalline Er^3+^ and Tm^3+^ co-doped MY(WO_4_)_2_, where M = Li, Na, K, double tungstates are reported. The obtained samples were characterized using standard X-ray powder diffraction (XRD) technique, Rietveld method, transmission electron microscopy (TEM), scanning electron microscopy (SEM) and IR spectroscopy. The smallest crystallites (about 13 nm) could be obtained for the sodium samples synthesized by both the Pechini (for the resin calcined at 550 °C) and hydrothermal methods (synthesis at 230 °C). The average particle size of nanocrystalline powders increases with increasing temperature. It was found that nanocrystals retain the bulk structure with tetragonal and monoclinic symmetry for the sodium and potassium analogues, respectively. In contrast to this behaviour, LiY(WO_4_)_2_ undergoes a size-induced structural transformation from monoclinic (space group *P*2/*n*) to tetragonal (space group *I*4_1_/*a*) symmetry. IR spectra of the synthesized sodium and potassium compounds are very similar to their bulk counterparts. IR spectra of the lithium analogues show, however, abrupt changes when the calcination temperature increases to 850 °C or higher. This behaviour is consistent with the size-induced phase transition in this compound.

## Abstract 2

TEM images of NaY(WO_4_)_2_:Er, Tm nanocrystalline powders synthesized by the Pechini method after calcination at (a) 550 °C, (b) 600 °C and (c) 700 °C.

## Graphical abstract

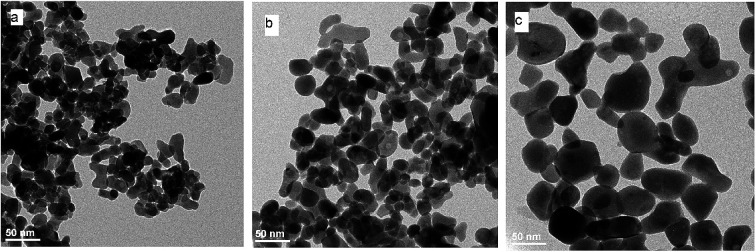

## Introduction

The MRE(WO_4_)_2_ (M = alkali metal ion, RE = lanthanide or Y ion) double tungstates have attracted a lot of attention due to their very good chemical stability, high optical absorption and transfer efficiency.^1^ They also exhibit strong stimulated Raman scattering due to large third-order electric susceptibility,^2,3^ and possess a great ability to accommodate various rare earth ions.^4^ All these features make them promising materials for solid-state laser applications.^1–4^ In recent years, growing interest has also been focused on developing synthesis methods of micro- and nanosized double tungstates since these materials in powder form appeared to be promising phosphors for visual displays and solid-state lighting.^3^

MRE(WO_4_)_2_ compounds have been reported to adopt either the scheelite- or wolframite-type structure, depending on the size of M and RE ions and/or the temperature of synthesis. The more recent review was made by Postema *et al.*^6^ It is well known that growing of very good optical quality single crystals of double tungstates or molybdates is the hard and long-standing process proceeded at very high temperature. Therefore, there is a scientific and technological interest in the developing of efficient methods for synthesis of ceramic materials with the well-controlled properties, like particle size, shape and crystal structure. It is also worth adding that many materials in the form of nano-sized crystallites can present new properties due to confinement effects and, in some cases, different structure compared to the bulk material.^7^

In the present paper, we report Pechini and hydrothermal synthesis and X-ray diffraction, electron microscopy and IR studies of nanocrystalline luminescent Er^3+^ and Tm^3^ co-doped Li/Na/KY(WO_4_)_2_ double tungstates. IR spectroscopy was used as tools for structural investigations since these techniques have been shown to be very useful in studies of nanocrystalline materials.^8^ We have chosen Er^3+^ and Tm^3+^ as active ions for doping due to their attractive characteristics for integrated optical applications. For instance, the 1.5 μm transition from Er^3+^ matches perfectly the third window defined for telecommunications whereas the emission at around 2 μm from Tm^3+^ has been used in optical amplification, surgery and remote sensing.^5^ It is worth adding here that in order to examine the luminescent properties of materials from the point of view of their applications, it is necessary to know the structure and physical properties of these materials because the photoluminescence behaviour is controlled by the local symmetry of an active ion and deviation from the average structure has been observed in many phosphors. The doping with thulium and erbium ions is not an important issue in this paper. However, it will be a subject of our future studies on luminescence properties of such nanocrystals. Such small difference in the amount of co-doped active ions is negligible for the structural measurements. Now, we present the elementary studies on our nanocrystals, *e.g.* the structure and dependence of the structure on the calcination temperature and type of alkali metal ion. Huang *et al.* investigated the effect of alkali-metal ions on the luminescence properties for alkali-metal europium double tungstates AEu(WO_4_)_2_ (A = Li, Na, K).^9^ However, this effect has not been well studied in case of yttrium-analogues. In particular, there are no reports concerning thulium doped monoclinic LiY(WO_4_)_2_ host. Thus, the main aim of the performed studies is analysis of the local structure and understanding the influence of alkali-metal type and crystallite size on structural and phonon properties of the examined materials what would help in more precise design of the luminescent materials.

## Experimental

The nanocrystalline Er^3+^ and Tm^3+^ co-doped M^I^Y^III^(WO_4_)_2_ tungstates, where M^I^ = Li, Na or K, were synthesized by both the Pechini and microwave-assisted hydrothermal methods. Commercially available chemicals of analytical grade were used without further purification.

In the Pechini synthesis, the experimental processing protocol used in the present work is similar to that described previously.^10^ It consists of two steps and no special equipment is necessary. At first, appropriate M_2_WO_4_·2H_2_O tungstate (Alfa Aesar, 99.5%) and (NH_4_)_6_W_12_O_41_ (Fluka), as well as Y(NO_3_)_3_·5H_2_O, Er(NO_3_)_3_·5H_2_O and Tm(NO_3_)_3_·5H_2_O nitrates (Alfa Aesar, 99.99%) were separately dissolved in the stoichiometric amounts of the deionised water. Next, the aqueous solution of citric acid was equally added to the above-prepared solutions. After complete homogenisation, both solutions were mixed together. Metal ions were chelated by the carboxyl groups of the citric acid and remained homogeneously distributed in the solution. To create a rigid polyester net, the appropriate amount of ethylene glycol was added (the molar ratio of the glycol to citric acid was 1 : 1). The mixture was dried with stirring at 80 °C for 20 h to reduce moisture, and then heated at 110 °C in the air atmosphere for 5 days. During this time, the solution turned into yellowish gel, which expanded several times from its original volume and, at the end, a brown resin was obtained. This resin, named as precursor, was rather hard and transparent for all tungstates. In the next step, precursor of each tungstate was divided into several samples. Each of them was calcined in air for 1 h at different selected temperature in the range 500–900 °C. During this procedure, the polymeric decomposition of the obtained resin occurred. Finally, each sample was cooled down to the room temperature. The light pink powders were obtained as final products.

The hydrothermal procedure does not need organic solvents and it can be concluded in a short time. In this synthesis, stoichiometric quantities of Y(NO_3_)_3_·5H_2_O, Er(NO_3_)_3_·5H_2_O and Tm(NO_3_)_3_·5H_2_O were dissolved in succession in a small amount of deionised water and mixed together. Separately, K_2_WO_4_·2H_2_O, Na_2_WO_4_·2H_2_O or Li_2_WO_4_·2H_2_O was also dissolved in deionised water. For each alkali metal, nitrate solution was mixed with the tungstate one under stirring. The obtained suspension was poured into a Teflon reaction vessel placed in a microwave autoclave (Magnum II autoclave – Ertec, Poland). Next, the suspension was preheated at 115 °C for half an hour. The hydrothermal reaction was subsequently carried on for three hours at isothermal condition at different temperatures. Maximum power of microwaves was 600 W. After this treatment, the reactor was cooled to room temperature. The as-obtained fine precipitates were centrifuged, washed several times with deionised water and dried in air at 80 °C for about 20 h. It is worth to adding that additional heating for an hour at 700 °C was required to obtain the desired potassium compounds.

The LiY(WO_4_)_2_ and NaY(WO_4_)_2_ tungstates were co-doped with the Er^3+^ and Tm^3+^ ions with the amount of 2% and 1%, respectively. The KY(WO_4_)_2_ tungstate was co-doped with higher amount of the same ions, *i.e.*, 5 and 2%, respectively. In terms of the simplicity of notation, the amount of dopants is not shown in the chemical formulas.

XRD characterization was carried out at room temperature by using an X'Pert PRO powder diffractometer (PANalytical, The Netherlands) working in the reflection geometry and using CuKα radiation (*λ* = 1.54056 Å) in step of 0.02° (2*θ*) in the 2*θ*-range between 10 and 80°. The analysis of each pattern was made by the LeBail fitting and Rietveld method using the FullProf Suite package, version September 2012.^11^ The profiles were described by a pseudo-Voigt function. The average crystallite sizes were evaluated by Williamson–Hall procedure^12^ for full set of Bragg reflections analysed individually by WinPlotr program implemented in FullProf Suite package (with parameter *K* = 1 and without any correction on the instrumental broadening of Bragg profile). For sake of comparison, the Scherrer values of crystallites size were also calculated without any corrections, and using *K* = 1.^13^ However, it is necessary to underline that the last values (although commonly published) are out of scientific value.^14^

The morphology and particle size of the selected samples of MY(WO_4_)_2_:Er, Tm were studied using TEM and SEM techniques. Specimens for TEM were prepared by dispersing a small amount of the nanocrystalline powder in pure ethyl alcohol with ultrasonic agitation. A droplet of the suspension was deposited on a copper microscope grid covered with carbon thin film. The grains on the supported films were then examined using conventional TEM (Tesla BS 500, operated at 90 kV). The microstructure was studied by high-resolution TEM (HRTEM, Philips CM20 SuperTwin operating at 200 kV). A FEI Nova NanoSEM 230 microscope equipped with an EDAX Pegasus XM4 spectrometer (with SDD Apollo 40 detector) was used to SEM microscopy.

Infrared spectra were measured with a Biorad 575C FT-IR spectrometer in KBr pellet in the 4000–400 cm^−1^ region and in Nujol suspension in the 500–50 cm^−1^ region. The spectral resolution was 2 cm^−1^. For the sake of comparison, the spectra of different powder samples were normalised to unity.

## Results and discussion

### X-ray powder diffraction, SEM and TEM studies of LiY(WO_4_)_2_:Er, Tm

Bulk LiY(WO_4_)_2_ undergoes phase transition at 988 K (715 °C) from the low-temperature monoclinic phase (space group *P*2/*c*) to another monoclinic phase (space group *P*2/*n*) and another phase transition at 1177 K (904 °C) into tetragonal phase (space group *I*4_1_/*a*).^15,16^ It should be noted that Postema *et al.*^6^ did not find the *P*2/*c* phase but transition into this phase was reported by Klevtsov and Kozeeva.^17^

**Fig. 1:**
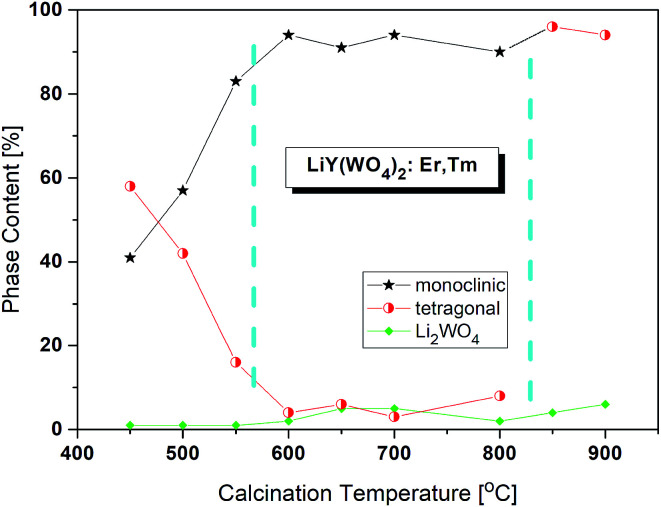
Changes of phase content in the samples calcined at different temperatures. The values are from the Rietveld analyses.

The X-ray powder diffraction patterns of the lithium samples obtained by the Pechini method at different temperatures are presented in Fig. S1a.† Three types of diffraction patterns can be distinguished corresponding to three calcination temperature regions (Fig. 1). The samples calcined up to about 575 °C contain a mixture of two LiY(WO_4_)_2_ phases, the monoclinic *P*2/*n* and tetragonal, in different proportions ranging from 41 : 58 to about 83 : 16. The second temperature region, from about 575 to 825 °C, corresponds to the nearly pure monoclinic phase. Only small percentage, about 4%, of the tetragonal phase can be observed. In the third temperature region, above 825 °C, the samples are tetragonal and the monoclinic phase totally disappeared. Unfortunately, all samples have also a small amount of trigonal Li_2_WO_4_ (about 1–7%).

As a starting data for consecutive Rietveld analyses, we used those for monoclinic LiY(WO_4_)_2_ (ICSD #261840 (ref. 6)) and tetragonal LiTb(WO_4_)_2_ (ICSD #261834 (ref. 6)), as well as Li_2_WO_4_ (ICSD #67236 (ref. 18)). The Rietveld refinement confirmed the structural models. An example of the results of such refinement of the X-ray diffraction patterns is presented in Fig. S2† and the detailed data (lattice parameters) from the Rietveld refinement are presented for selected samples in Table 1. The resulting accuracy (*e.g.* Bragg *R*-factor, *R*_B_) seems to be quite reasonable for the structure refinement of nanocrystalline samples. The lattice parameters of the both main phases are plotted in Fig. S3.† It is important to emphasize that the arrangement of structural units in the monoclinic and tetragonal phases is quite different, that is, the monoclinic phase (*P*2/*n*) is constructed from a zig-zag chains of WO_6_ octahedra sharing edges to form [W_2_O_8_] ribbons going along the *c*-direction (*c* ≈ 5 Å), while the tetragonal phase (*I*4_1_/*a*) contains separated WO_4_ tetrahedra. Thus, the molecular mechanism of the structural phase transition is not clear.

**Table 1:** The results of Rietveld refinement for selected samples of LiY(WO_4_)_2_:Er, Tm. Data for Li_2_WO_4_ are in bottom part of the table

		500 °C	650 °C	900 °C
*I*4_1_/*a*	Part of sample [%]	42	6	92
	*a* [Å]	5.1527(2)	5.1695(6)	5.1518(1)
	*c* [Å]	11.1261(6)	11.1214(26)	11.1453(2)
	*V* [Å^3^]	295.40(2)	297.21(9)	295.81(1)
*P*2/*n*	Part of sample [%]	56	89	0
	*a* [Å]	9.9884(4)	9.9917(3)	—
	*b* [Å]	5.7872(2)	5.7915(1)	—
	*c* [Å]	5.0061(2)	5.0064(1)	—
	*β* [°]	94.222(2)	94.205(1)	—
	*V* [Å^3^]	288.58(2)	288.92(1)	—
*R*3̄	Part of sample [%]	2	4	8
	*a* [Å]	14.359(5)	14.361(1)	14.361(2)
	*c* [Å]	9.714(5)	9.608(1)	9.613(3)
	*γ* [°]	120	120	120
	*V* [Å^3^]	1734(2)	1716(0)	1717(1)

It is interesting to note that the *c*-parameter of Li_2_WO_4_ undergoes a great jump at about 575 °C (from 9.70 to 9.60 Å) what can be a sign of size-induced phase transition (Fig. S4†). However, the trigonal symmetry, characteristic for the high-temperature phase of the bulk sample, is preserved at least down to 450 °C. Thus, we do not observe the orthorhombic phase existing for the bulk material below 573 K.^16,19^

**Fig. 2:**
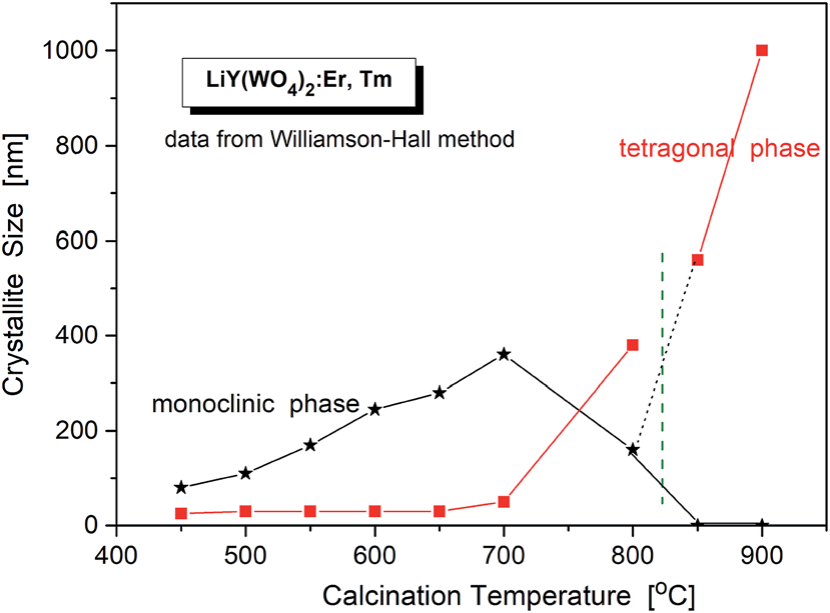
Crystallite size of different phases in the samples of LiY(WO_4_)_2_:Er, Tm *vs.* calcination temperature.

The critical crystallite diameter for the phase transition from the monoclinic to tetragonal phase can be evaluated as about 400 nm (Fig. 2). Thus, we observe the so-called size-induced structural phase transition (see below). Moreover, the tetragonal phase has stable small crystallites (of about 30–50 nm) over a wide range of the calcination temperature (from 450 °C to 700 °C), while monoclinic grains grow up to about 400 nm at 700 °C. Distribution of the particle sizes obtained from the Scherrer calculations is rather large, *i.e.*, small particles have size of about 30–40 nm and the large ones – 160–400 nm.

Another interesting feature of the studied nanocrystals is presence of the re-entrant size-induced phase transition with respect to the tetragonal phase. That is, the same tetragonal phase is observed below 575 °C and above 825 °C. This thermal behaviour of LiY(WO_4_)_2_:Er, Tm nanocrystals is very interesting because such sequence of phases is rarely observed for nanocrystalline materials. In general, nanocrystalline materials exhibit the inverse sequence of phase transitions,^7^*i.e.*, from a higher-symmetry phase to lower-symmetry one when the crystallites grow up from nanosized to microsized. In other words, a high-temperature phase can be stabilized for nanosized crystallites but large, microcrystalline crystallites should have the same symmetry as the bulk material. In our case this must be the monoclinic *P*2/*n* that is observed for the bulk LiY(WO_4_)_2_ below 1177 K (904 °C). However, in our experiment, the grains obtained after calcination at 850 and 900 °C have tetragonal symmetry. Presence of a re-entrant phase transition is uncommon for nanocrystals, and described as type C2 in the classification of size-induced phase transitions.^7^ There are not so many crystals with such type of phase transition diagram, only 17 with respect to 417 known size-induced phase transitions.^20^

**Fig. 3:**
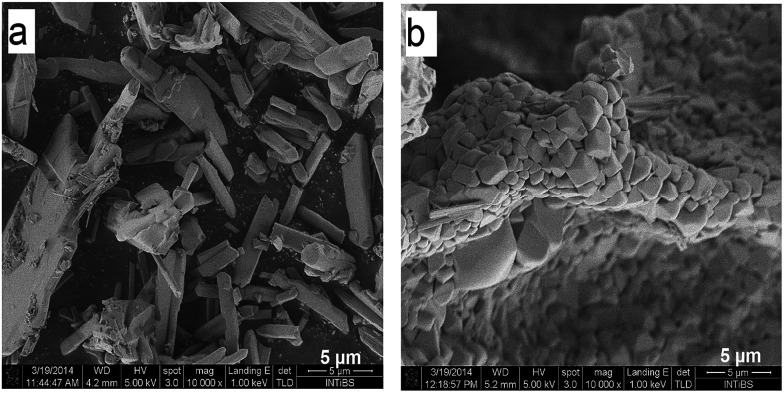
SEM images of the LiY(WO_4_)_2_:Er, Tm samples calcined at 700 °C (a) and 850 °C (b).

**Fig. 4:**
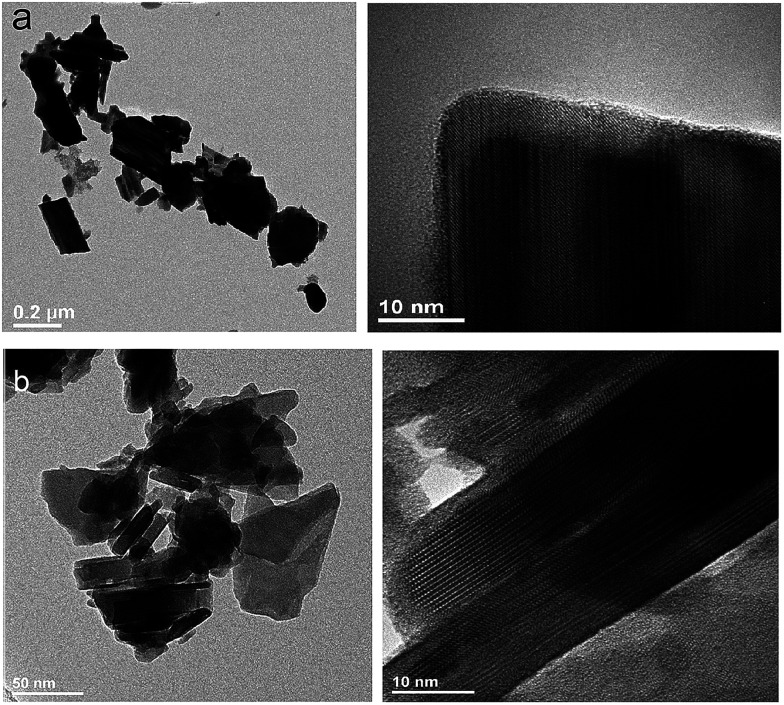
TEM and HRTEM micrographs of the (a) LiY(WO_4_)_2_:Er, Tm samples calcined at 750 °C during Pechini method and (b) LiY(WO_4_)_2_:Er, Tm obtained from the hydrothermal method at 230 °C.

To obtain detailed information on morphology of crystallites synthesized by both the Pechini and hydrothermal method and monitoring the microstructure evolution with increase of calcination temperature SEM and TEM observations were carried out. Fig. 3 shows SEM micrographs of the samples calcined at 700 °C and 850 °C. The sample calcined at 850 °C is mainly composed of tetragonal bipyramidal particles with size of 1 μm. EDS analysis at various points showed that the sample is homogeneous, and composition of the grains (Y – 13.4 at%, Er – 0.4 at%, Tm – 0.2 at%, W – 21 at%, O – 65 at%) fits rather well the expected one. It should be noted that Li content in the sample could not be measured due to the technical limitations of this method. EDS analysis confirms also that dopant ions (Er, Tm) are incorporated into the crystal structure. When the calcination temperature decreases to 700 °C, the shape of nanoparticles significantly changes, they have mainly thin plate shape and the crystallite size distribution is not homogeneous, *i.e.*, both the grains smaller than 100 nm appear and some of them are large, up to 750 nm. Further information on size and morphology of the synthesized crystallites was obtained by using transmission electron microscopy (TEM). Representative micrographs (TEM and HRTEM) for the sample synthesized by the Pechini method at 750 °C is shown in Fig. 4a. The distribution of particle size is large, it varies from about 40 to 400 nm. On the other hand, TEM images of the lithium sample obtained at 230 °C using the hydrothermal method (Fig. 4b) show very thin plates with lateral size and thickness from about 12–16 nm to 30–50 nm, respectively. The distribution of particle size is much narrower (see Fig. S5a†). The high resolution HRTEM images (Fig. 4) show clear lattice fringes, indicating highly crystalline nature of LiY(WO_4_)_2_ particles.

### X-ray powder diffraction, SEM and TEM studies of NaY(WO_4_)_2_:Er, Tm

NaY(WO_4_)_2_ is a member of the tungstate family with the scheelite (CaWO_4_) structure of *I*4_1_/*a* symmetry, where the Ca^2+^ ions are statistically substituted by Na^+^ and Y^3+^ ions. X-ray powder diffraction (Fig. S1b†) shows that all XRD patterns measured for our nanocrystalline samples correspond well to the pattern of the reference tetragonal NaY(WO_4_)_2_ with the unit cell parameters *a* = *b* = 5.205, *c* = 11.282 Å, and *V* = 305.6 Å^3^ (*I*4_1_/*a*, ICSD #192112).^21,22^ According to literature data, NaY(WO_4_)_2_ does not exhibit any phase transition below its melting temperature (1483 K).^23^ As this compound is a laser host crystal, several papers were devoted to properties of the samples doped with various ions (see ref. 24 and the literature therein). Herein, we report studies of samples of previously not reported chemical compositions, *i.e.*, containing erbium and thulium dopants. Moreover, our samples were prepared in the nanocrystalline form.

X-ray powder diffraction patterns of samples prepared by the Pechini and the hydrothermal synthesis show the presence of the same diffraction lines (Fig. S1b†). The diffraction lines are significantly broadened for the samples prepared by the Pechini method and this broadening decreases when the calcination temperature increases from 550 °C to 900 °C. This result indicates that the obtained samples are nanocrystalline and that the size of nanocrystallites increases with increasing calcination temperature. Broadening of diffraction lines is also visible for the samples synthesized by the hydrothermal method, but it weakly depends on the synthesis temperature. Obtained results indicate that the formation of NaY(WO_4_)_2_ starts at about 550 °C and 200 °C for the Pechini and hydrothermal method, respectively.

**Fig. 5:**
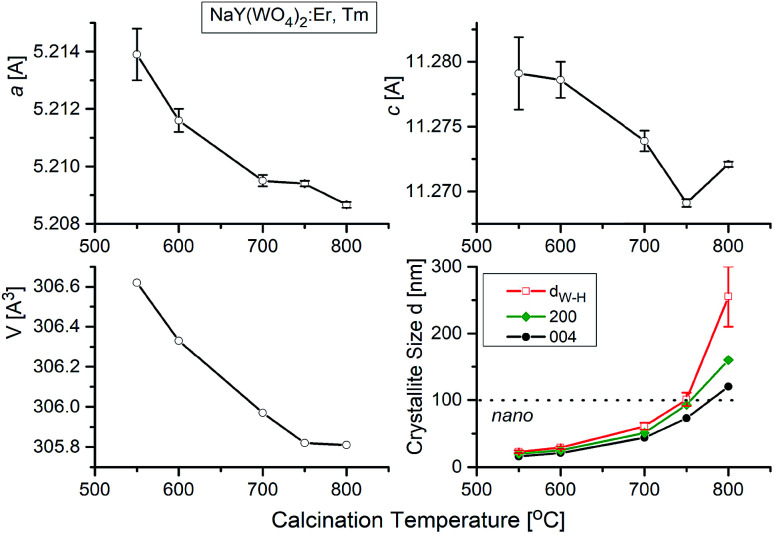
Lattice parameters and crystallite size *vs.* calcination temperature for NaY(WO_4_)_2_:Er, Tm. The different lines of temperature dependence of crystallite size correspond to data from Williamson–Hall approximation (upper line) and from Scherrer calculations using 200 and 004 Bragg lines, respectively (bottom lines).

The temperature dependences of the lattice parameters are presented in Fig. 5. As can be seen, the lattice parameters decrease with the calcination temperature, what is characteristic for nanocrystalline matter. The crystallite size of each sample, *d*_W–H_, were calculated by Williamson–Hall method without any correction for instrumental broadening. Fig. 5 shows that the crystallites grow up from about 15 nm for the sample calcined at 550 °C to about 260 nm for the sample treated at 800 °C. We also show for the comparison sake the crystallite sizes calculated using Scherrer formula (*K* = 1, without corrections) for two main Bragg lines: 200 and 004. These data show similar dependence on the calcination temperature and there is no indication on the size-induced phase transition.

**Fig. 6:**
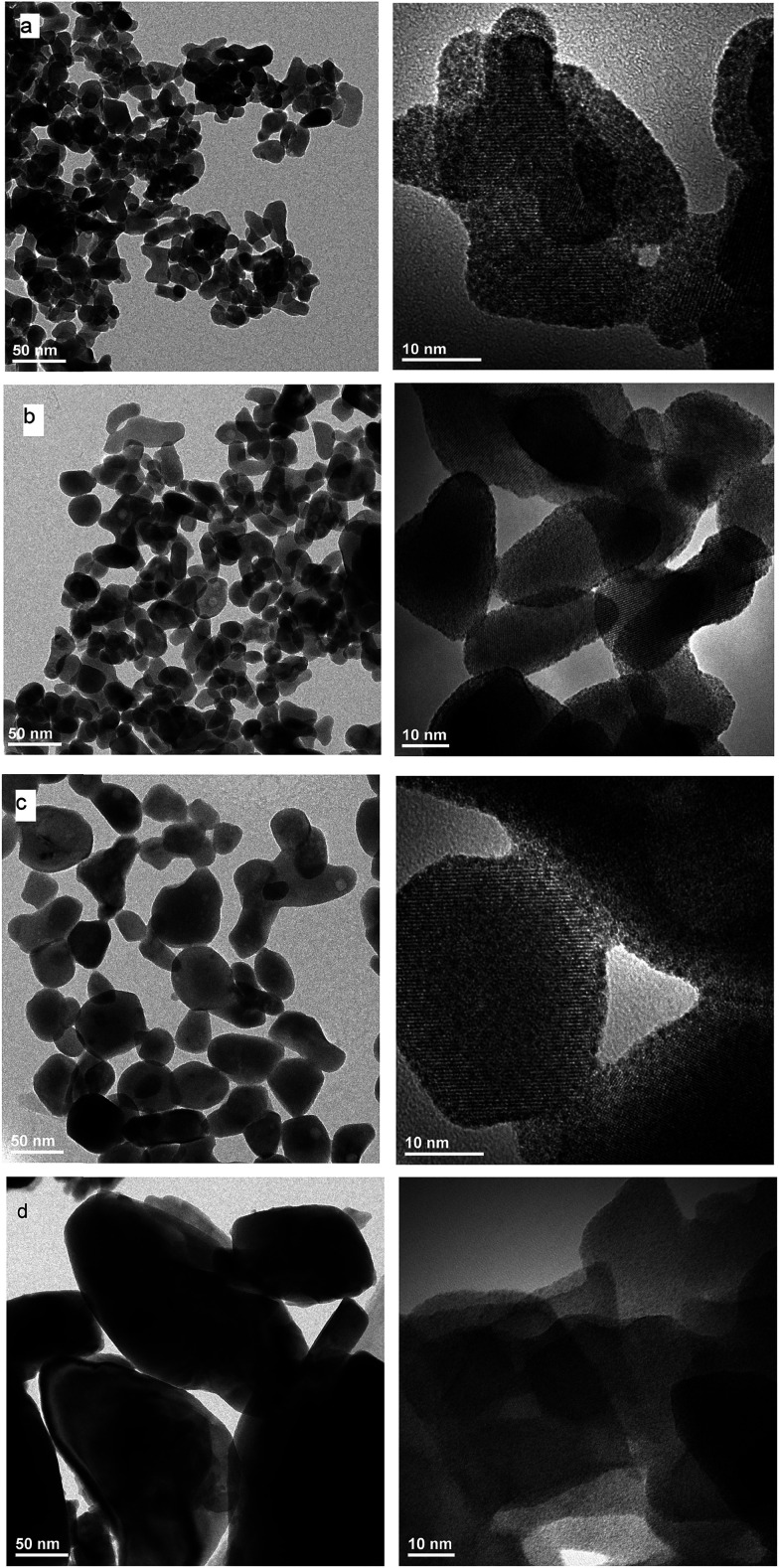
TEM and HRTEM images of NaY(WO_4_)_2_:Er, Tm calcined at (a) 550 °C, (b) 600 °C and (c) 700 °C and (d) NaY(WO_4_)_2_:Er, Tm obtained from the hydrothermal method at 250 °C.

EDS analysis at various points showed that the sample is homogeneous, and composition of the grains (Na – 11 at%, Y – 10 at%, Er – 0.6 at%, Tm – 0.2 at%, W – 23 at%, O – 55 at%) fits rather well the expected one. EDS analysis confirms also that dopant ions (Er, Tm) are incorporated into the crystal structure. The transmission electron microscopy (TEM) images (Fig. 6) reveal that the morphology of grains for sodium double tungstate samples is homogenous. The HRTEM image shows the presence of well-defined atomic planes, confirming the highly crystalline nature of these particles. Particles become more oval with increase of the calcination temperature. Distribution of the particle size is relatively small, especially for the samples obtained by the Pechini method. Size of the non-agglomerated nanoparticles with a slightly elongated shape is around 13–14, 20 and 30 nm for the calcination temperature at 550 °C, 600 °C and 750 °C, respectively. These results agree well with the estimated crystallite size evaluated from the XRD line broadening. The experimental conditions of the hydrothermal method at 250 °C allow obtaining particles with a bigger size, *i.e.*, about 60 nm. In this case, the particle size distribution is not so narrow, *i.e.*, one can observe particles both with about 20 and with about 100 nm size (Fig. S5b†).

### X-ray powder diffraction, SEM and TEM studies of KY(WO_4_)_2_:Er, Tm

Bulk KY(WO_4_)_2_ crystallizes in two modifications: low-temperature monoclinic scheelite-type (space group *I*2/*c*; *a* = 8.0, *b* = 10.3, *c* = 7.5 Å, *β* = 94.4°; unconventional setting) and high-temperature monoclinic phase (space group *P*2_1_/*c*; *a* = 17.4, *b* = 10.3, *c* = 7.8, *β* = 94.6°).^25–27^ However, recently Bhat *et al.* synthesized a high-temperature tetragonal phase of KNd(WO_4_)_2_ nanoparticles by a solution combustion route.^28^ It is worth adding that the low-temperature monoclinic phase can also be described in another, conventional unit cell (*a* = 10.6, *b* = 10.3, *c* = 7.5 Å, *β* = 130°, space group *C*2/*c*; ICSD #90378).^29,30^ The axis transformations can be achieved by *a*′ = (*a* + *c*), *b*′ = *b*, *c*′ = *c*, where *a*′, *b*′ and *c*′ are for the *I*2/*c* phase. The phase transition, associated with change in the oxygen coordination of W atoms from six- to fourfold, occurs at about 1240 K (1027 °C).^16,30,31^

The X-ray powder diffraction of studied KY(WO_4_)_2_ samples shows that the samples are biphasic for low-temperature of calcination and monophasic for the calcination temperature higher than 670 °C (Fig. S1c†). The phase corresponding to larger crystallites, prepared at high calcination temperatures, is monoclinic and the same as the room-temperature phase of bulk material (*I*2/*c*). At low calcination temperatures, this monoclinic phase coexists with another phase of unknown symmetry. This results points to existence of a size-induced phase transition of very popular type B2. The supposition that this new phase is the same as the high-temperature monoclinic phase of bulk KY(WO_4_)_2_ does not work. The diffraction pattern extracted from the data measured for the sample calcined at 550 °C (where only small admixture of the low-temperature monoclinic phase is observed) seems to be clearly established (Fig. S1c†). The indexation by LeBail fitting gives the monoclinic unit cell of *a* = 9.7273(14), *b* = 7.7352(7), *c* = 5.1118(4) Å and *β* = 105.11(2)°, and the possible symmetry is *P*2_1_. This unit cell is different from that of the high-temperature phase of bulk KY(WO_4_)_2_ but similar to the unit cell reported for nanocrystalline KY(WO_4_)_2_ doped with Ho^3+^ and Yb^3+^ ions.^25^ Unfortunately, the attempts to solve the crystal structure was not successful. Our results also show that the samples prepared by the hydrothermal method have the same structure sequence as the samples prepared by the Pechini method.

**Fig. 7:**
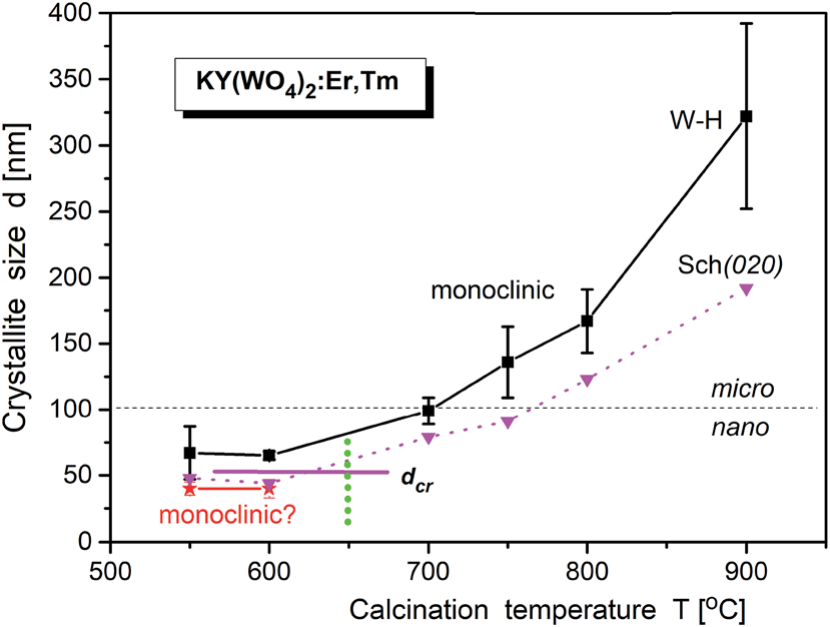
Temperature dependence of crystallite size of KY(WO_4_)_2_:Er, Tm (from Pechini synthesis) calculated by Williamson–Hall method. The error bars correspond to the standard deviations of mean Williamson–Hall approximation of crystallite size. The vertical dashed line, as well as horizontal line, indicate on the region of the size induced phase transition (temperature and critical size, respectively). The data from Scherrer calculations (from 020 Bragg line) are also presented (as triangles).

LeBail fitting procedure for nanocrystalline KY(WO_4_)_2_:Er, Tm calcined at 900 °C gave the lattice parameters *a* = 8.0828(2), *b* = 10.3365(3), *c* = 7.5483(2) Å and *β* = 94.363(2)°; *V* = 628.03(3) Å^3^. Fig. S6† shows that these parameters decrease with increase of the calcination temperature. The crystallite size, calculated by WilliamsonHall method (*d*_W–H_), increase for the main monoclinic phase from about 50 nm to 280 nm, respectively, when the calcination temperature increases from 550 °C to 900 °C (Fig. 7). The second, minor *P*2_1_ phase exists up to the calcination temperature of about 650 °C and has crystallites of about 40 nm size. Thus, one can suppose that the size-induced transition occurs at about 50 nm. Note, that the presented values of the crystallite size are only approximate (qualitative and overestimated) due to lack of any corrections used and systematic errors impossible to evaluate. However, the relative changes with the calcination temperature correctly show the temperature behaviour.^14^

**Fig. 8:**
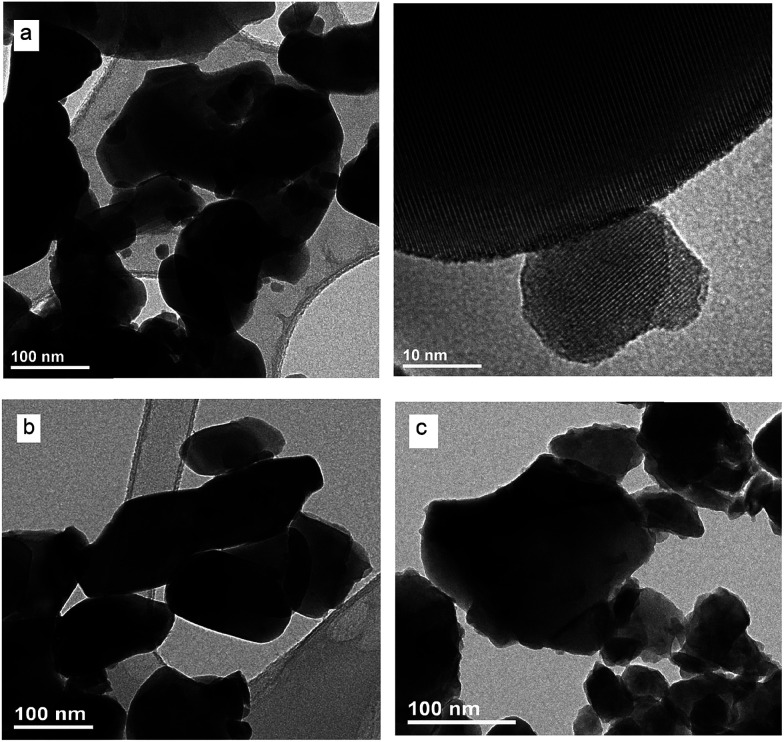
TEM and HRTEM images of KY(WO_4_)_2_:Er, Tm calcined at (a) 700 °C and (b) 750 °C and (c) KY(WO_4_)_2_:Er, Tm obtained from the hydrothermal method at 250 °C and heated subsequently at 700 °C for an hour.

EDS analysis at various points showed that the sample is homogeneous, and composition of the grains (K – 10 at%, Y – 9 at%, Er – 0.7 at%, Tm – 0.3 at%, W – 22 at%, O – 58 at%) fits rather well the expected one. EDS analysis confirms also that dopant ions (Er, Tm) are incorporated into the crystal structure. The homogeneity of grain morphology is rather poor as can be seen in the TEM images (Fig. 8). Oval and plate-type particles can be seen in the samples obtained by the Pechini method while plates are characteristic for the samples obtained by the hydrothermal method. The particle size distribution is narrower for the samples obtained by the hydrothermal method and mean size of the non-agglomerated nanoparticles is around 29 nm (see Fig. S5c†).

### Infrared studies

**Fig. 9:**
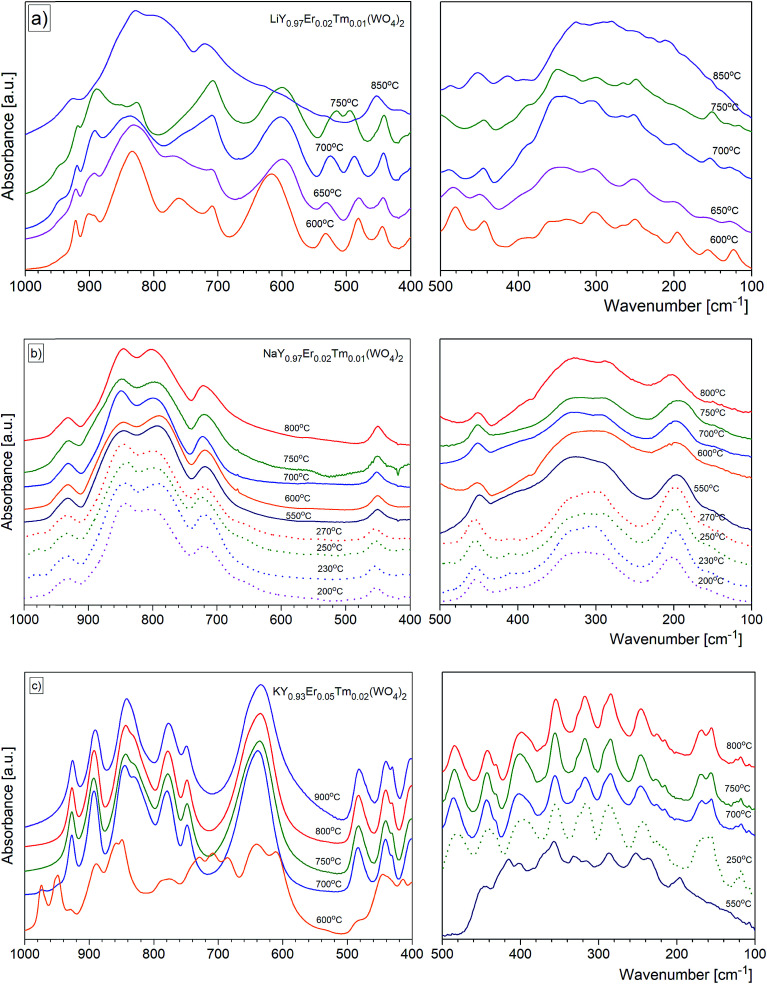
The evolution of mid_IR and far_IR spectra by the calcination temperature for the (a) LiY(WO_4_)_2_:Er, Tm, (b) NaY(WO_4_)_2_:Er, Tm and (c) KY(WO_4_)_2_:Er, Tm nanopowders obtained by the Pechini (solid line) and hydrothermal (broken line) methods.

For most nanomaterials, IR spectra remain sufficiently similar to those corresponding to single crystal, facilitating direct identification of the phases. The same behaviour is observed for our compounds synthesized by the Pechini and hydrothermal methods. The recorded IR spectra as a function of the calcination temperature (Pechini method) or synthesis temperature (hydrothermal method) are presented in Fig. 9. Table S1† lists wavenumbers of the observed modes.

Previous results of lattice dynamics calculations as well as polarized IR and Raman scattering studies gave a detailed insight into vibrational properties of rare earth double tungstates.^32–34^ In principle, IR spectra consist of two separated regions: 700–930 cm^−1^ for the bands assigned to the stretching modes and 100–500 cm^−1^ for those assigned to the bending and lattice modes. This situation is characteristic for the well-isolated WO_4_ tetrahedral units, as in case of NaY(WO_4_)_2_:Er, Tm nanopowders. Our results show that the bands at 930, 845, 801 and 720 cm^−1^ can be attributed to symmetric and asymmetric stretching vibrations of the W–O bonds within the WO_4_ units. The corresponding bending vibrations are located at about 450, 330 and 285 cm^−1^. The presence of bands specific for the tetragonal symmetry, unchanged with the calcination temperature (Fig. 9b) implies that the structure of all studied NaY(WO_4_)_2_:Er, Tm samples does not change.

In the case of KY(WO_4_)_2_:Er, Tm nanopowders one can observe narrowing of the separation between bands characteristic for the stretching and bending modes (Fig. 9c). This behavior can be attributed to the fact that the WO_4_ units are no longer isolated. Indeed, the crystal structure with monoclinic symmetry contains double chains of W_2_O_10_ units connected by double oxygen bridges and extended along the crystallographic *c*-axis. Single oxygen bridges connect these units along the crystallographic *a*-axis. The bands corresponding to the stretching modes of the double and single oxygen bridges appear at about 890, 777, 749 and 635 cm^−1^, and those related to the bending modes, at about 484, 398, 355, 326 and 285 cm^−1^. The translational and librational modes of K^+^, Ln^3+^ and WO_4_^2−^ ions are observed in the region below 260 cm^−1^. Translational modes of the metal ions are strongly coupled and can be described as mixed T′(K^+^, W^6+^) and T′(Ln^3+^, W^6+^) translational lattice modes. IR bands of the KY(WO_4_)_2_:Er, Tm nanopowders reflect the monoclinic structure and remain unchanged for the samples calcined in the 700–900 °C range. This behavior is consistent with the XRD data that show large size of the crystallites in these samples (more than 100 nm).

**Fig. 10:**
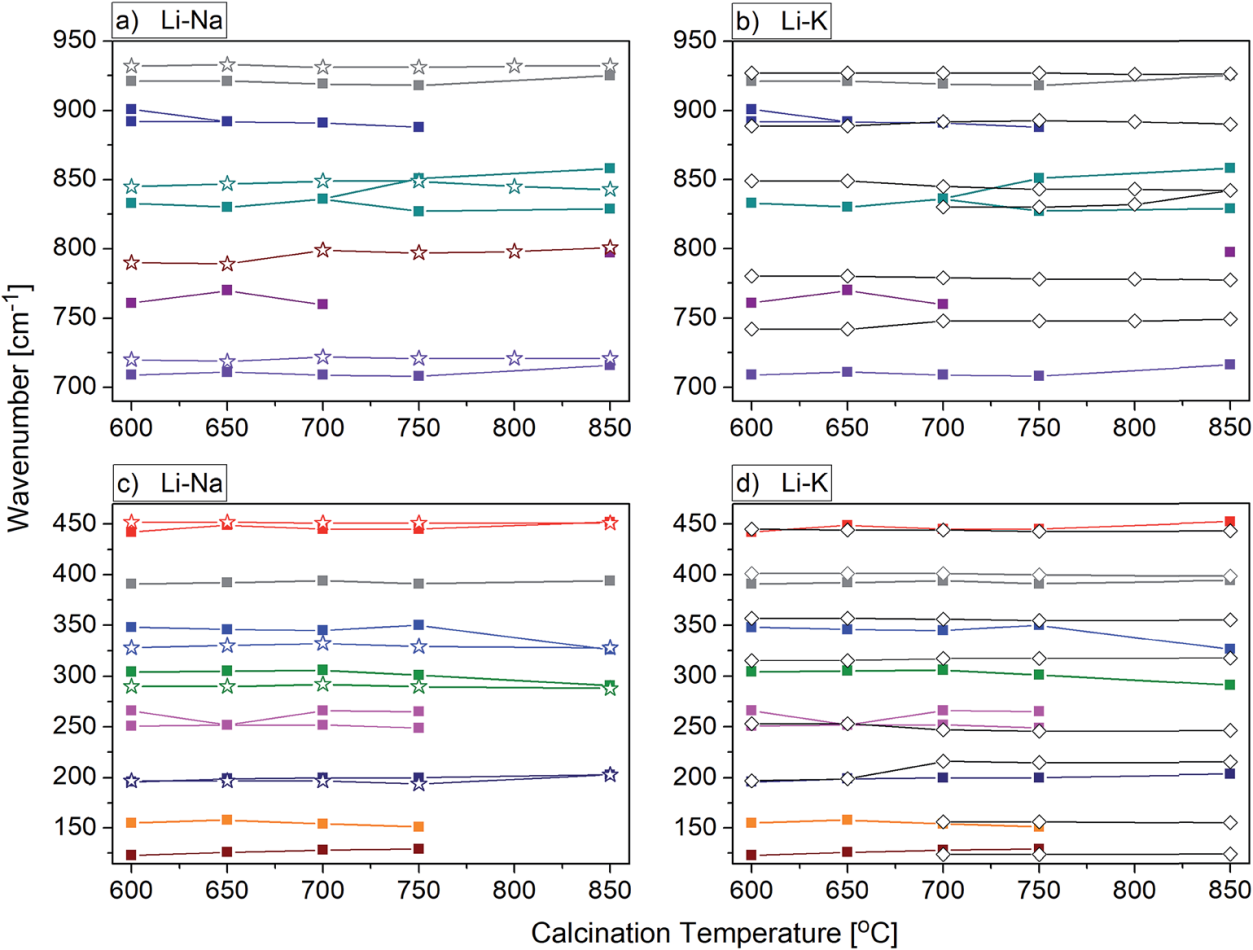
Calcination temperature dependence of the infrared phonon modes for the LiY(WO_4_)_2_:Er, Tm, NaY(WO_4_)_2_:Er, Tm and KY(WO_4_)_2_:Er, Tm nanopowders obtained by the Pechini method. Fig. 10a and b show the range of stretching modes and Fig. 10c and d show the range of bending modes and lattice vibrations. Data of the lithium samples are signed by the solid squares (a–d), sodium – by the empty stars (a and c) and potassium – by the empty rhombi (b and d).

Behaviour of the lithium samples is significantly different than behaviour of the sodium and potassium analogues. First of all, the lithium-based samples exhibit a size-induced phase transition from the monoclinic to tetragonal symmetry near 850 °C and this transition is clearly seen through abrupt changes in the spectra measured for the samples annealed at 750 and 850 °C (Fig. 9a). In particular, the IR spectrum recorded for the sample calcined at 850 °C shows significantly smaller number of bands compared to the spectrum recorded for the sample calcined at 750 °C (see Fig. 9a and 10). Furthermore, the observed bands are significantly broader. The energy range of the stretching and bending vibrations extends from 515 to 948 cm^−1^ and 266 to 500 cm^−1^, respectively, for the compounds with the monoclinic structure. The corresponding regions for the compounds with the tetragonal structure are much narrower, *i.e.*, 716–925 cm^−1^ for the stretching and 290–487 cm^−1^ for the bending modes. These changes are consistent with change of the tungsten-oxygen coordination to tetrahedral.

## Conclusions

We have successfully synthesized, using Pechini and hydrothermal methods, nanocrystalline LiY(WO_4_)_2_, NaY(WO_4_)_2_ and KY(WO_4_)_2_ double tungstates co-doped with Er^3+^ and Tm^3+^ ions. The type of alkali metal has significant impact on the calcination temperature at which the desired crystalline phase of the double tungstate can be obtained. The minimum synthesis temperature is 550 °C, 550 °C and 670 °C for the lithium, sodium and potassium samples, respectively.

Detailed structural properties of the obtained double tungstate samples were characterised by the X-ray powder diffraction, TEM, SEM and IR spectroscopy. The analysis of X-ray powder diffraction diagrams confirms that the compounds were obtained as single phases. The average grain size of these samples increases with increasing calcination temperature. According to the Williamson–Hall calculations, the smallest size of crystallites (15 nm) is obtained for the sodium-containing samples calcined at 550 °C (Pechini method). The hydrothermal crystallization process allows obtaining plate-like crystallites of very small thickness (down to 15 nm) for LiY(WO_4_)_2_:Er, Tm and NaY(WO_4_)_2_:Er, Tm.

Vibrational properties depend on the nanoparticle size due to presence of defects and the phonon confinement effect. In addition, the lithium compounds exhibit a size-induced phase transition from the monoclinic to tetragonal symmetry. We have shown that it is possible to obtain the tetragonal scheelite-like phase in pure form (at 850 °C or above) for the lithium compound. Such tetragonal phase could not be obtained by using other reported methods.^6^ We have also shown that type of alkali metal ion has a rather strong effect on the phonon properties of double tungstate family of compounds. We have shown that the phonon properties of MY(WO_4_)_2_:Er, Tm are significantly different when compared compounds with different alkali metal. This behaviour proves that the alkali metal ions have significant influence on the phonon and structural properties of this family of compounds. This behaviour is much more noticeable in the group of Li^+^, Na^+^ and K^+^ ions than when potassium compound compared to rubidium one, as proved by Mączka *et al.*^35^ by comparison of phonon properties of KNd(WO_4_)_2_ and RbNd(WO_4_)_2_ double tungstates. Type of alkali metal ions has also significant effect on morphology of the synthesized samples, as revealed by TEM and SEM data.

In summary, we report the facile, rapid and low-cost method even for the large-scale production of alkali-metal rare earth double tungstate nanocrystals. The results indicate that the conditions of syntheses can be an effective tool influencing structural and phonon properties of the double tungstates. We show, for instance, that the Pechini method is the most suitable especially for synthesis of pure samples containing relatively small NaY(WO_4_)_2_ nanocrystallites. The proposed methods might be quite easily adopted for technological applications.

## Conflicts of interest

There are no conflicts to declare.

## Supplementary Material

RA-008-C7RA10706D-s001
