# Supplementary material for: Alkali metal impact on structural and phonon properties of Er3+ and Tm3+ co-doped MY(WO4)2 (M = Li, Na, K) nanocrystals
Source: RSC Adv. 2018 Jan 10;8(5):2632–41. doi: 10.1039/c7ra10706d (PMC9077457; doi:10.1039/c7ra10706d)
Supplement: RA-008-C7RA10706D-s001 [file RA-008-C7RA10706D-s001.pdf]

Electronic Supplementary Material (ESI) for RSC Advances.
This journal is © The Royal Society of Chemistry 2018

Supporting Information

Alkali metal impact on structural and phonon properties of Er^{3+} and Tm^{3+} co-doped $MY(WO_4)_2$ (M = Li, Na, K) nanocrystals

Paulina Ropuszyńska-Robak, Paweł E. Tomaszewski, Leszek Kępiński, Lucyna Macalik

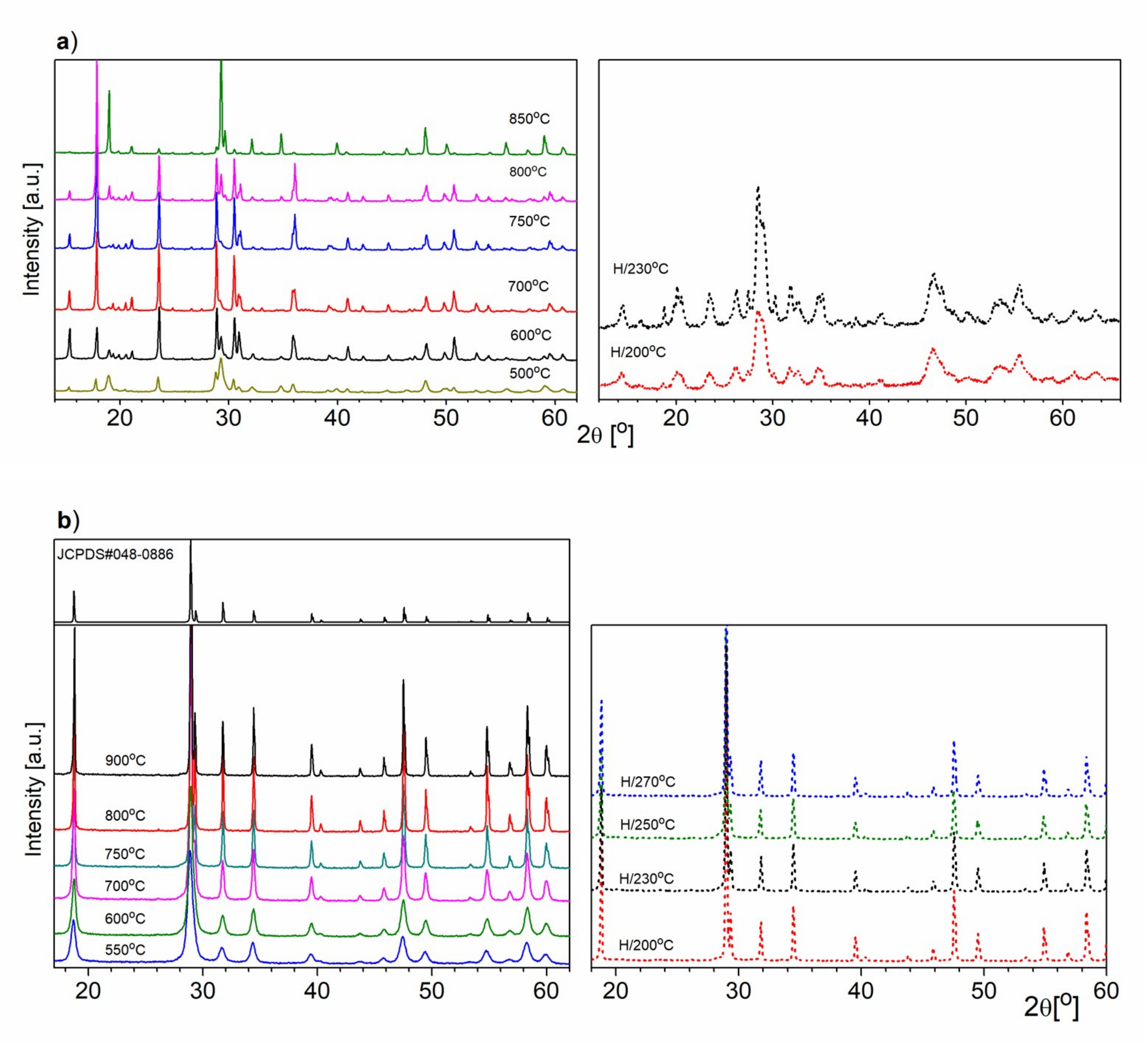

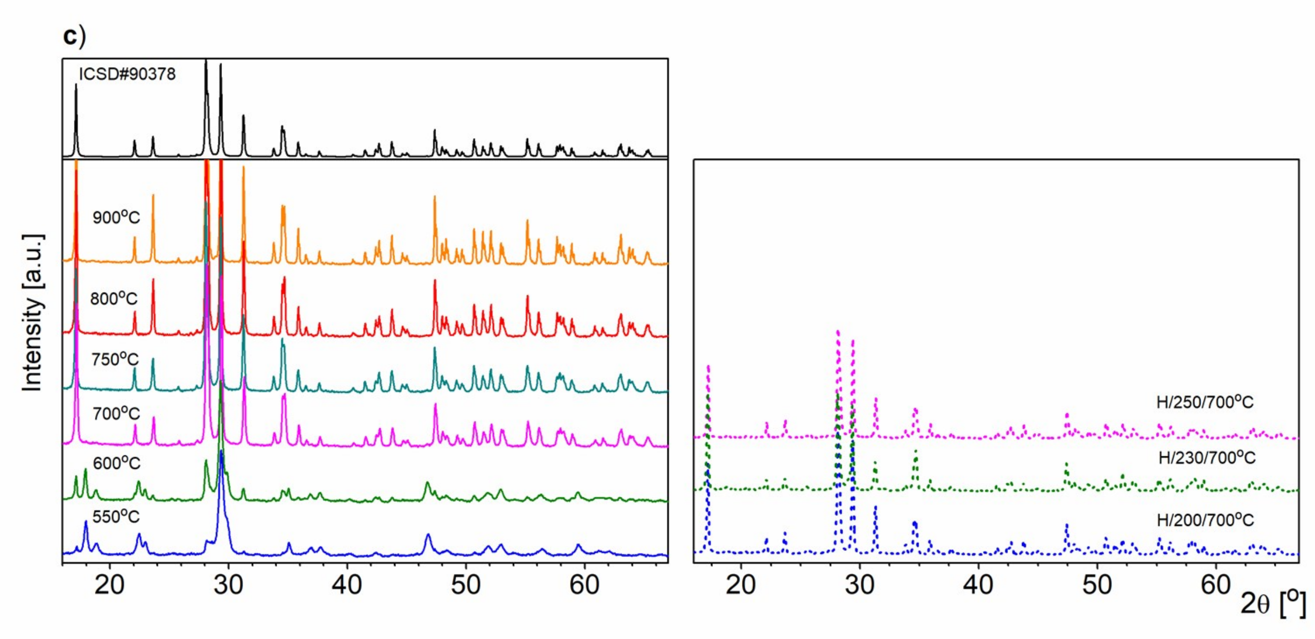

Figure S1. The selected diffraction patterns of (a) $LiY(WO_4)_2$:Er,Tm, (b) $NaY(WO_4)_2$:Er,Tm and (c) $KY(WO_4)_2$:Er,Tm nanopowders obtained by the Pechini method calcined at various temperatures (solid line) and by the hydrothermal method (broken line). Diffraction pattern of the standard monoclinic [E. Gallucci, C. Goutaudier, M.T. Cohen-Addad, B.F. Mentzen, T. Hansen, J. Alloys Compd. 306 (2000) 227] and tetragonal [Y. He, G. Wang, Z. Luo, Chin. Phys. Lett. 10 (1993) 667] phases are added for comparison.

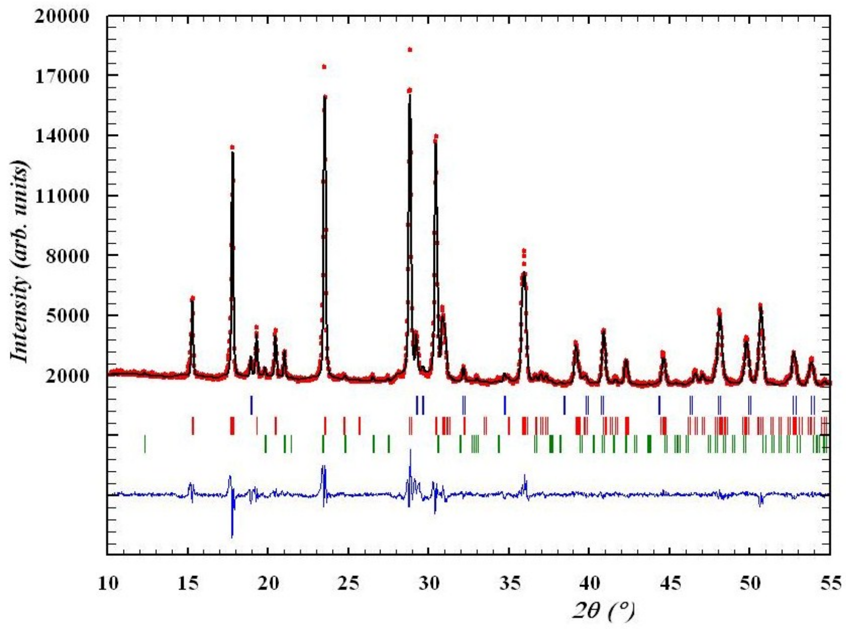

Figure S2. . Final Rietveld plot for the sample of $LiY(WO_4)_2$:Er,Tm calcined at 600°C. The circles are the experimental values; the continuous lines stand for the calculated pattern. Vertical bars correspond to the position of Bragg peaks of tetragonal structure (upper line), monoclinic

phase (middle line) of $LiY(WO_4)_2$ and of Li_2WO_4 (bottom line). The bottom curve represents the difference between experimental and calculated diffraction patterns.

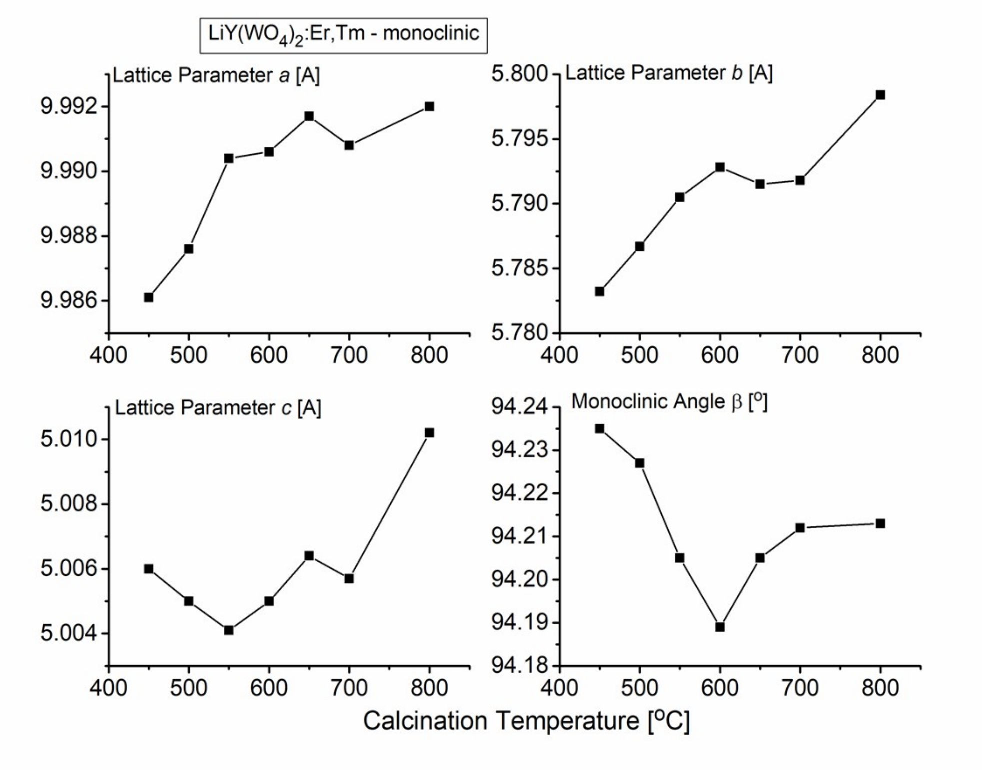

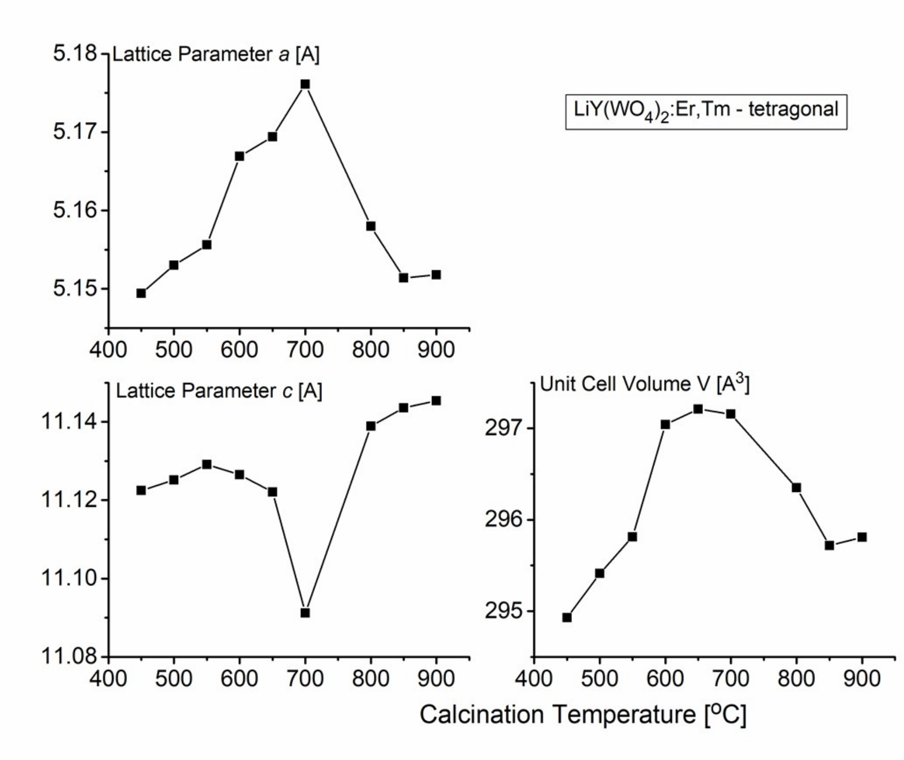

Figure S3 Lattice parameters vs. calcination temperature for (a) monoclinic and (b) tetragonal phase.

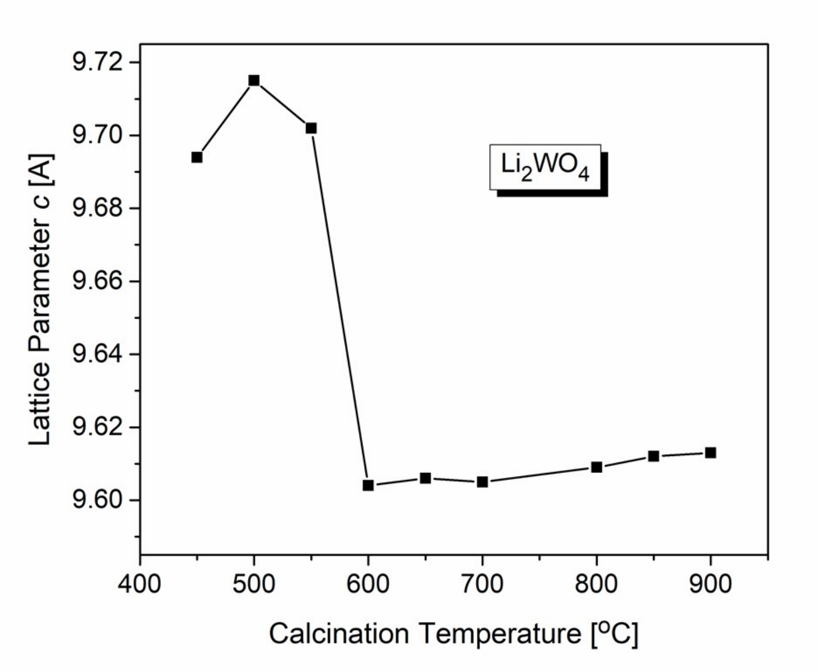

Figure S4. Lattice parameter c of Li_2WO_4 nanocrystals vs calcination temperature.

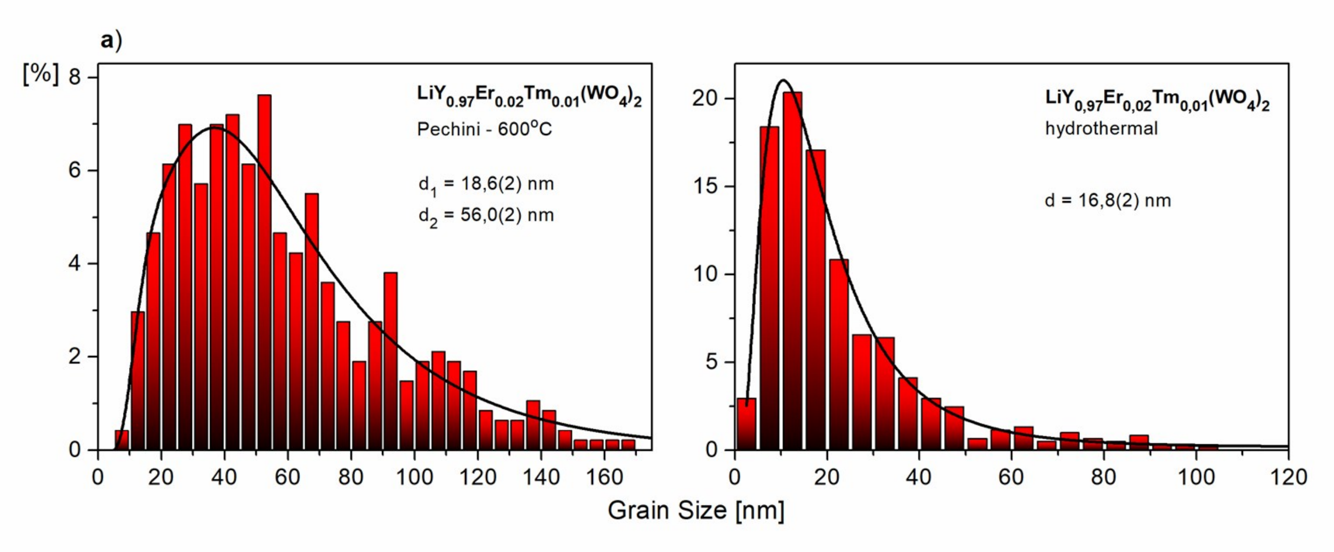

b)

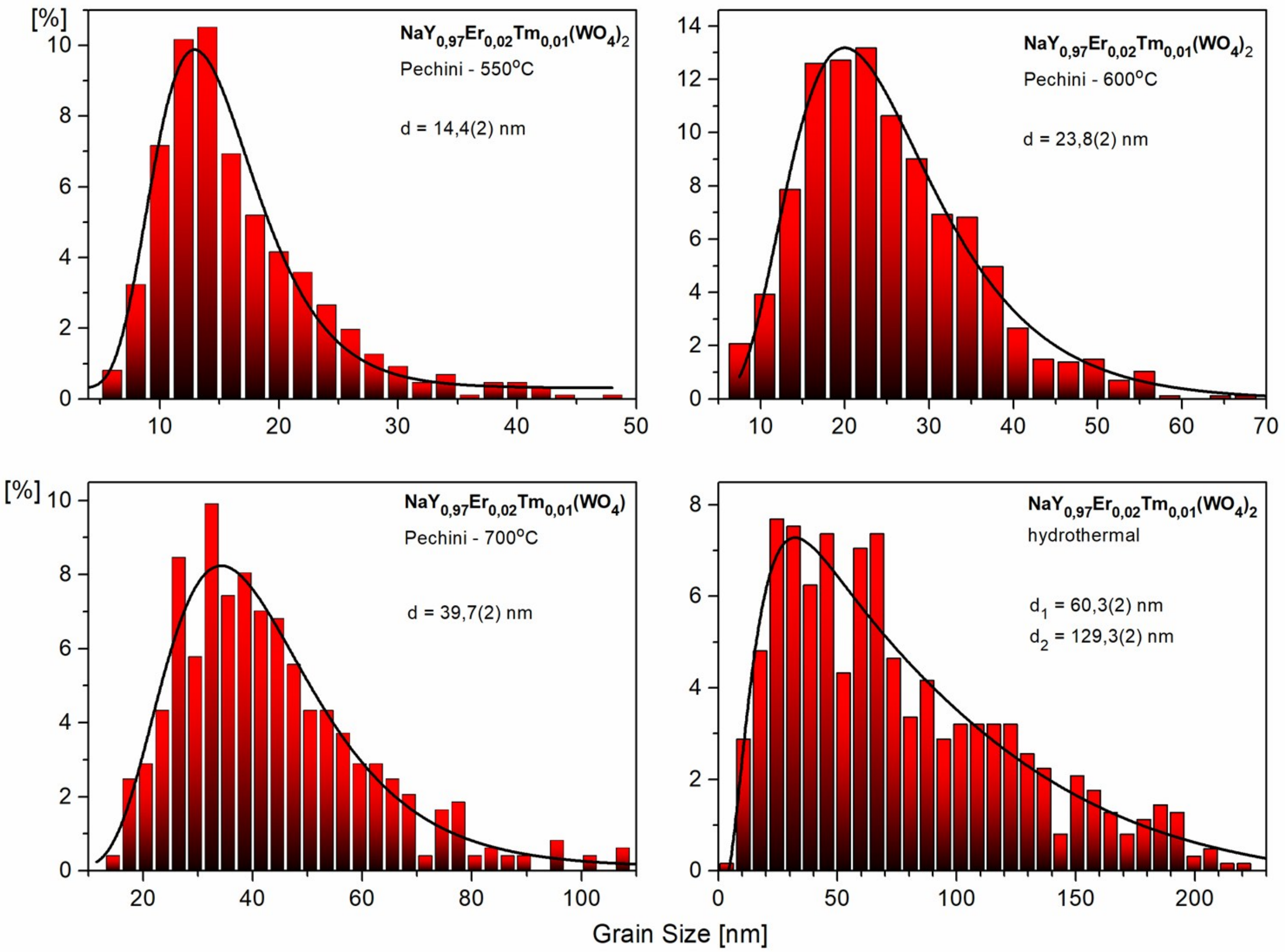
[%]
$NaY_{0,97}Er_{0,02}Tm_{0,01}(WO_4)_2$
Pechini - 550°C
d = 14,4(2) nm
$NaY_{0,97}Er_{0,02}Tm_{0,01}(WO_4)_2$
Pechini - 600°C
d = 23,8(2) nm
[%]
$NaY_{0,97}Er_{0,02}Tm_{0,01}(WO_4)$
Pechini - 700°C
d = 39,7(2) nm
$NaY_{0,97}Er_{0,02}Tm_{0,01}(WO_4)_2$
hydrothermal
d_1 = 60,3(2) nm
d_2 = 129,3(2) nm
Grain Size [nm]

c)

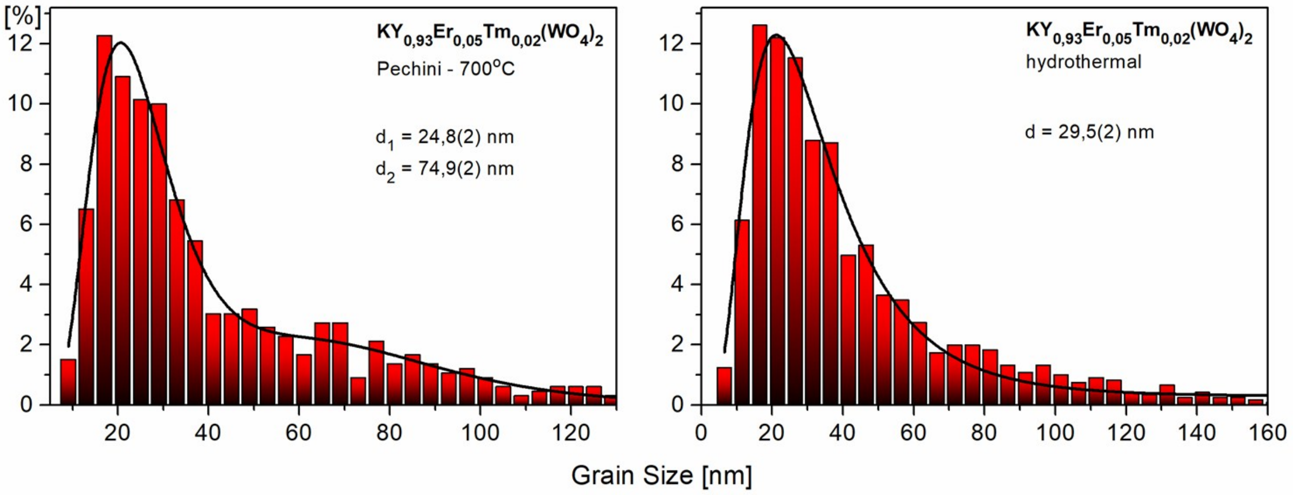
[%]
$KY_{0,93}Er_{0,05}Tm_{0,02}(WO_4)_2$
Pechini - 700°C
d_1 = 24,8(2) nm
d_2 = 74,9(2) nm
$KY_{0,93}Er_{0,05}Tm_{0,02}(WO_4)_2$
hydrothermal
d = 29,5(2) nm
Grain Size [nm]

Figure S5. Histograms of particle size distribution of (a) $LiY(WO_4)_2$:Er,Tm, (b) $NaY(WO_4)_2$:Er,Tm and (c) $KY(WO_4)_2$:Er,Tm nanopowders. The fitting curves represent double-peak LogNormal approximation.

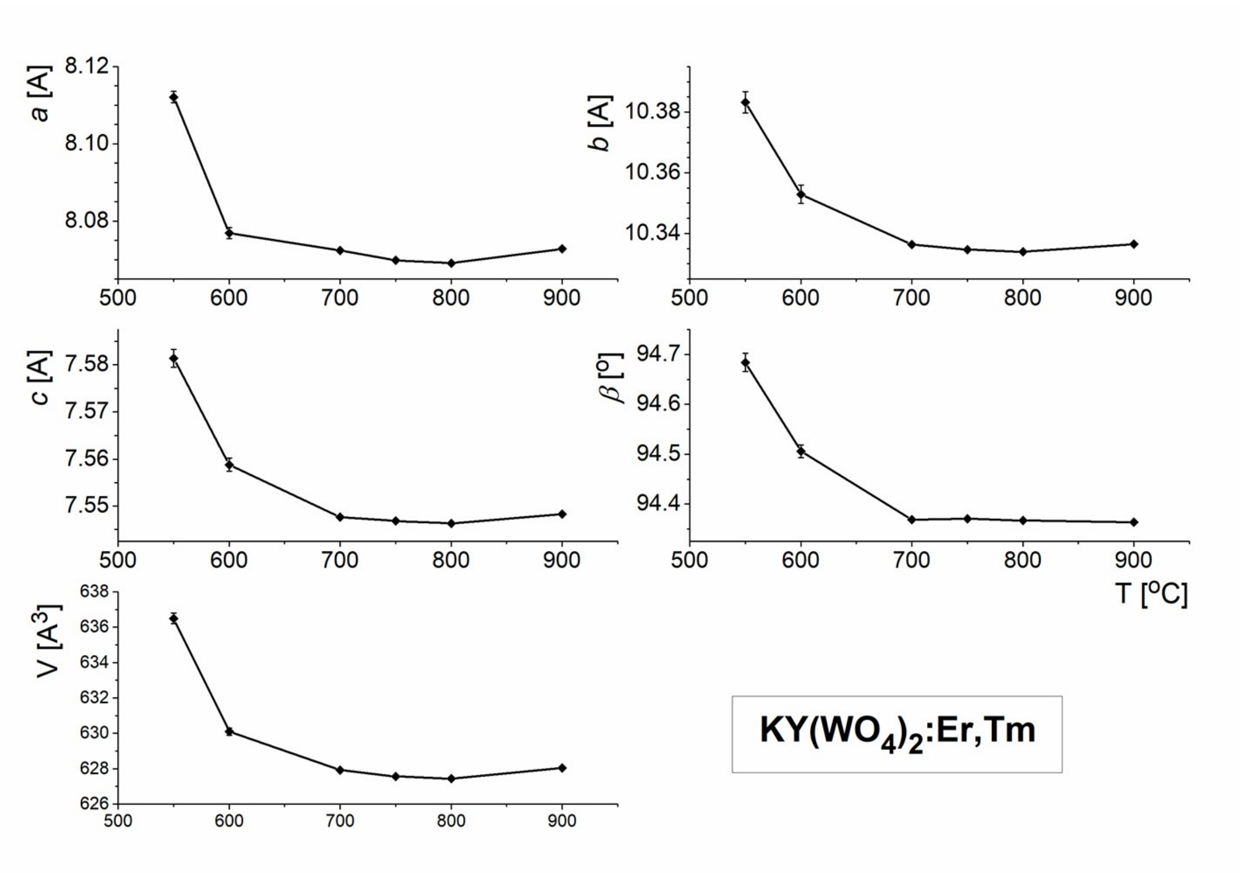

Figure S6. Lattice parameters of the main phase of $KY(WO_4)_2$:Er,Tm nanocrystals (from Pechini synthesis) vs. calcination temperature.

Table S1. Wavenumbers of IR bands observed for the synthesized samples.

Li					Na					K				Assignment
600 C	650 C	700 C	750 C	850 C	600 C	650 C	700 C	750 C	850 C	600ºC	700ºC	750ºC	850ºC	
										974m				unidentified
942sh	948sh	946sh	947sh							949m				ν(W-O)
921m	921m	919m	918m	925sh	932w	933w	931w	931w	932w	927w	927m	927m	926m	ν(W-O)
901m										889s	892s	893s	890m	ν(WOW)
892sh	892m	891s	888vs							858vs				ν(WOW)
			851sh	858sh	845s	847s	849vs	849vs	845vs	849vs	845s	843s	842s	ν(W-O)
833vs,b	830vs,b	836vs,b	827s	829vs,b	790vs	789vs	799s	797s	801vs		830sh	830sh		ν(W-O)
761m,b	770m,b	760sh		797sh						780w,b	779s	778s	777m	ν(WOOW)
											748m	748m	749m	ν(WOOW)
				716s	720m	719m	722m	721m	721m	729m				ν(W-O)
709m	711m	709s	708vs							708m				ν(W-O)
										685m	639vs,b	636vs,b	635vs,b	ν(WOOW)
616vs,b	599s,b	602s,b	599vs,b							641m				ν(WOOW)
532w	531w	525m	515m							610m	485m	484m	484m	ν(WOOW)
480m	484m	489m	497sh	487sh						483sh				ν(WOOW)
442m	449m	445m	445m	453m						445m	444m	443m	443m	δ(WOOW)
				413w	452w	452w	451w	451w	451w	415m	431sh	431sh	431sh	δ(W-O)
391w		394sh	391sh							401m	401m,b	400m,b	398m,b	δ(WOOW)
										370sh	356m	355m	355m	δ(WOW)
348m,b	346s,b	345s,b	350s,b							357m	326sh	326sh	326sh	and
										332w	317m	317m	317m	δ(W-O)
				326s,b	328m,b	330m,b	332m,b	329m,b	328m,b	315w	293sh	293sh	293sh	δ(W-O)
303m	305s	307s	301m	291s,b	289m,b	290m,b	290m,b	290m,b	285m,b					δ(W-O)
266sh		267sh	266sh							286w	285m	285m	285m	δ(WOOW)
										253m	247m	246m	246m	lattice modes
250w	252m	252m	249m							235m	227w	227w	226w	lattice modes
231sh				208m,b						197w	216w	215w	215w	lattice modes
197w	200m	200w			197w	197w	197w	194w	202w		169m	169m	169m	T’(M^+/Ln^{3+})
155w	158w	154w	151w								156m	156m	155m	lattice modes
122w	128w	128w									124w	124w	124w	lattice modes
116sh											118w	118w	119w	T’(WO_6)

(abbreviatons: vs – very strong, s – strong, m – medium, w – weak, vw – very weak, sh – shoulder, b – broad)
